# Comparison of the clinical and MRI features of patients with hepatic hemangioma, epithelioid hemangioendothelioma, or angiosarcoma

**DOI:** 10.1186/s12880-020-00465-4

**Published:** 2020-06-29

**Authors:** Zhuangsheng Liu, Lilei Yi, Junhao Chen, Ruqiong Li, Keming Liang, Xiangmeng Chen, Ronggang Li, Wansheng Long

**Affiliations:** 1 Department of Radiology, Jiangmen Central Hospital, Affiliated Jiangmen Hospital of Sun Yat-Sen University, No. 23 Haibang Street, Jiangmen, 529000 Guangdong China; 2 Department of Radiology, Foshan Hospital of Traditional Chinese Medicine, Foshan, Guangdong China; 3 Department of Pathology, Jiangmen Central Hospital, Affiliated Jiangmen Hospital of Sun Yat-Sen University, Jiangmen, Guangdong China

**Keywords:** Liver, Hemangioma, Epithelioid hemangioendothelioma, Angiosarcoma, Magnetic resonance imaging

## Abstract

**Background:**

Comparisons of hepatic epithelioid hemangioendothelioma (HEHE), hepatic hemangioma, and hepatic angiosarcoma (HAS) have rarely been reported. The purpose of our study was to analyze the clinical and magnetic resonance imaging (MRI) findings of these conditions.

**Methods:**

A total of 57 patients (25 with hemangioma, 13 with HEHE, and 19 with HAS) provided hepatic vascular endothelial cell data between June 2006 and May 2017.

**Results:**

The proportions of cases with circumscribed margins were 88% (22/25), 84.6% (11/13), and 31.6% (6/19) for hemangioma, HEHE, and HAS, respectively (*P* < 0.001). HAS lesions were less likely to have circumscribed margins. The proportions of lesions with hemorrhaging were 4% (1/25), 30.8% (4/13), and 36.8% (7/19) for hemangioma, HEHE, and HAS, respectively (*P* = 0.014). HEHE and HAS cases were more likely to show heterogeneous signals on T1-weighted (T1WI) MRI. HEHE and HAS cases were more likely to show heterogeneous signals on T2-weighted (T2WI) MRI. Centripetal enhancement was the most common pattern in vascular tumors, with proportions of 100, 46.2% (6/13), and 68.4% (13/19) for hemangioma, HEHE, and HAS, respectively. The difference in enhancement pattern between HEHE and HAS was not significant, but rim enhancement was more common for HEHE (46.2%, 6/13).

**Conclusions:**

Our study revealed clinical and imaging differences between HEHE and HAS. The platelet count (PLT) and coagulation function of the HAS group decreased, whereas the alpha-fetoprotein (AFP) level increased. The 5-year survival rate for HAS was significantly lower than that of HEHE. A higher malignancy degree indicated a more blurred lesion margin, easier occurrence of hemorrhaging, and more heterogeneous T1WI and T2WI signals.

## Background

Liver-derived vascular endothelial cells can cause angiogenic lesions, including those that are benign (hepatic hemangioma), low-grade (hepatic epithelioid hemangioendothelioma, HEHE), or malignant (hepatic angiosarcoma, HAS). The clinical treatment and prognosis of these conditions vary greatly [1–4]. HEHE and HAS are rare and poorly understood liver malignancies [5–7] with low incidences. Their preoperative diagnoses require invasive puncture, which can lead to complications such as hemorrhaging and biliary fistula. Therefore, reliable and noninvasive preoperative imaging is necessary. Ultrasound, computed tomography (CT), magnetic resonance imaging (MRI), and positron emission tomographic PET-CT have been reported in the diagnosis of HEHE and HAS [8–12] in a few cases [10, 11, 13]. However, comparisons of HEHE and hemangioma with HAS have rarely been reported. It is important to distinguish HEHE from other liver tumors because long-term survival can be achieved with appropriate treatments such as surgical resection, liver transplantation, chemotherapy, or radiation. The 5-year survival rate of HAS is significantly lower than those of HEHE and hemangioma. Therefore, it is important for radiologists to be familiar with the imaging manifestations of HEHE and HAS. In this study, MRI findings and the differences among HEHE, hemangioma and HAS were compared and analyzed to improve the preoperative MRI diagnosis rates of HEHE and HAS.

## Methods

### Clinical data

This retrospective study was conducted with the approval of our hospital’s institutional review board, and all cases were obtained from a picture archiving and communication system (PACS). Cases of hepatic hemangioma, HEHE, and HAS between June 2006 and May 2017 were collected. All patient diagnoses were confirmed by biopsy or surgical resection, and all patients underwent preoperative contrast enhanced MRI. This study included 57 patients with tumors derived from hepatic vascular endothelial cells: 25 with hemangioma, 13 with HEHE, and 19 with HAS. The male-to-female ratio of the sample was 24:33, and the median age was 48 years (17–80 years). None of the patients had a history of exposure to cesium dioxide, arsenic, or other toxins. Additional disease history and laboratory test results are shown in Table 1.

**Table 1:** The clinical features of hepatic hemangioma, epithelioid hemangioendothelioma and angiosarcoma

Features	Hemangioma	HEHE	HAS	P
	(*n* = 25)	(*n* = 13)	(*n* = 19)	
Age(midian, range)	45 (24–62)	48 (21–80)	53 (17–71)	0.148
Sex(male: female)	11:14	5:8	8:11	0.950
Liver lesions				<0.001
single	4	1	4	
multiple	21	12	15	
Size (cm)				0.001
Mean ± SD	8.60 ± 3.60	4.28 ± 2.06	9.13 ± 4.20	
Range	4.1–18.2	1.3–7.0	3.6–18	
Blood platelet				
normal	25	11	14	
high	0	2	0	
low	0	0	5	
Blood coagulation function				0.299(HEHE vs HAS)
normal	25	11	13	
abnormal	0	2	6	
AFP				0.625(HEHE vs HAS)
normal	25	12	15	
high	0	1	4	
CEA (normal)	25 (100%)	13 (100%)	19 (100%)	
Cirrhosis	0	3	7	0.467(HEHE vs HAS)
Hepatitis B	2	5	3	<0.001
Excision	25	12	11	<0.001(HEHE vs HAS)

*FLL* focal liver lesions; *HEHE* hepatic epithelioid hemangioendothelioma; *HAS* hepatic angiosarcoma

### MRI scan

A GE Optima MR360(General Electric Company, US) was used with a field strength of 1.5 T and an eight-channel abdominal surface coil. Before scanning, the patients fasted for 4 h and were trained to breathe. The patients were positioned supine on the examination bed, feet first. A sagittal T2-weighted image (T2WI) was acquired using a fat-suppressed fast spin-echo sequence (TR/TE: 7059/85 ms; field of view: 44 × 40 cm; matrix: 320 × 224; thickness: 8 mm). An axial T1-weighted image (T1WI) was also acquired (TR/TE: 190/4.3 ms, slice thickness: 8 mm, image matrix: 256 × 160, field of view: 44 × 40 cm).

Gadopentetate dimeglumine (Magnevist, Schering, Berlin, Germany) was intravenously injected before obtaining the fat-suppressed transverse T1WIs. The dose was 0.1 mmol/kg per patient, and the injection rate was 2.0 ml/s (TR/TE: 3.6/1.7 ms, slice thickness: 5 mm, image matrix: 256 × 192, field of view: 40 × 44 cm). The scanning delay times were 22 s, 60 s, and 180 s for the arterial, portal, and delay phases, respectively.

### Image analysis

Two radiologists who had performed liver imaging diagnoses for more than 10 years analyzed all MR signs and determined whether the lesion margins were circumscribed and whether hemorrhage had occurred as well as observed the signal intensity and MRI enhancement pattern (i.e., centripetal enhancement, rim enhancement, septal enhancement, or no enhancement). The types of margin included circumscribed and not circumscribed, where the latter refers to peritumoral buds on enhanced scans. Concentric enhancement means that the peripheral nodular irregular post-contrast enhancement on the early phase, which progresses centripetally on delayed phase. Rim enhancement means that the enhancement is more pronounced at the periphery of the lesion. Septal enhancement represents a linear or reticular enhancement within the lesion. The radiologists were blind to clinical and pathologic data.

### Pathological analysis

Specimens were fixed in 10% formaldehyde solution for 24 h, dehydrated, paraffin-embedded, sectioned, conventionally stained with hematoxylin and eosin (H&E), and immunohistochemically stained using the streptavidin-peroxidase (S-P)-linked method via DAB staining. A senior pathologist specializing (21 years of working experience) in the diagnosis of liver tumors read the specimens.

### Statistical analyses

All clinical and imaging characteristics of the three lesion types were categorized and are listed in Tables 1 and 2. The data were analyzed using SPSS 24.0. The measurement data were represented as means±SDs. Between-group comparisons were performed using the one way ANOVA. Count data were represented as frequencies or rates, and the chi-square tests were performed. Differences with *P* < 0.05 were considered significant.

**Table 2:** The MRI features of hepatic hemangioma, epithelioid hemangioendothelioma and angiosarcoma

manifestations	Hemangioma	HEHE	HAS	t values/chi-square values	*P* value
boundary				17.949	<0.001
clear	22	11	6		
unclear	3	2	13		
hemorrhage				7.963	0.014
Yes	1	4	7		
No	24	9	12		
Signal on T1WI				11.271	0.004
homogeneous low	18	6	4		
heterogeneous low	7	7	15		
Signal on T2WI				14.638	0.001
homogeneous high	16	3	2		
heterogeneous high	9	10	17		
Enhancement				24.420	<0.001
centripedal filling	25	6	13		
rim	0	6	2		
septal	0	1	1		
none	0	0	3		

*HEHE*hepatic epithelioid hemangioendothelioma; *HAS*hepatic angiosarcoma

## Results

### General information and follow up

As Table 1 shows, this study included 57 patients (25 with hemangiomas, 13 with HEHE, and 19 with HAS) with tumors derived from hepatic vascular endothelial cells. No significant differences were found with regard to age of onset (*P* = 0.148) or sex (*P* = 0.950) among the three patient groups. The cases of HAS were associated with liver cirrhosis(*P* = 0.001). The liver lesions manifested mostly as masses, with proportions of 84, 92.31, and 78.95% for hemangiomas, HEHE, and HAS, respectively. The differences among these groups were significant (*P* < 0.001). The average tumor diameter was significantly higher (9.13 ± 4.20 cm, *P* = 0.001) in the hepatic hemangiosarcoma group than those in the hepatic hemangiomas and hepatic epithelioid hemangioendothelioma (8.60 ± 3.60 cm and 4.28 ± 2.06 cm, respectively). The surgical resection rates were 100% (25/25), 92.31% (12/13), and 57.89% (11/19) for hemangioma, HEHE, and HAS, respectively. The HAS resection rate was the lowest, with a significant difference of *P* < 0.001. The proportions of patients with hepatitis in the three groups were 8% (2/25), 38.46% (5/13), and 15.79% (3/19) for hemangioma, HEHE, and HAS, respectively (*P* = 0.063). The survival time was tracked for 8–39 months. The 13 cases of HEHE in this study showed no lung and bone metastases during the follow up after hepatectomy. Eleven patients in the HAS group died, but no deaths occurred in the hemangioma or HEHE groups.

### MRI findings

**Fig. 1:**
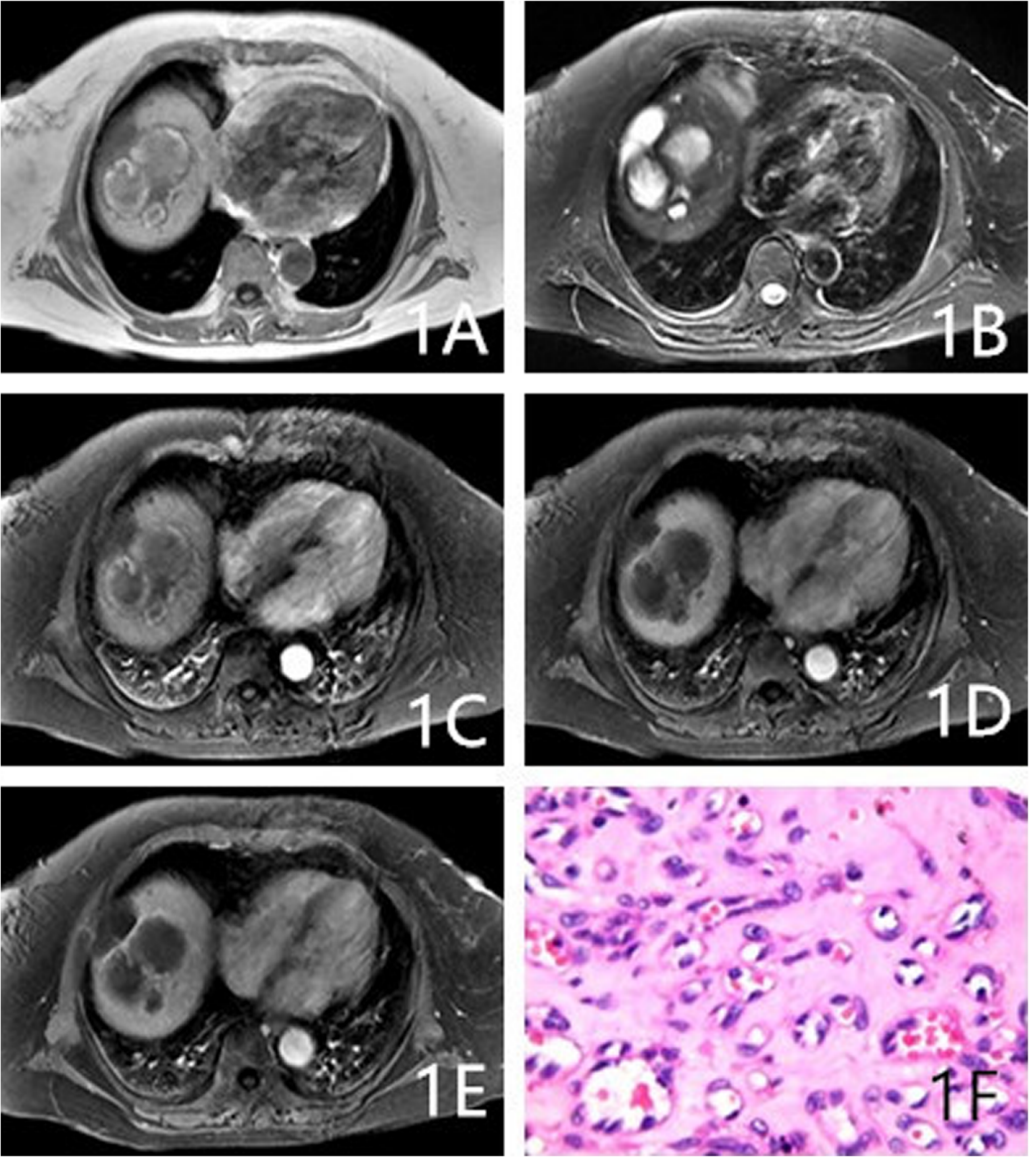
An 80-year-old female had multiple HEHE lesions in the right hepatic lobe. **a-e**: Multiple heterogeneous encapsulated masses were observed on T1WI and T2WI. The enhanced scan showed rim enhancement in the arterial, portal, and delayed phases. F: Magnification 400×. Lumens of different sizes in the interstitium and RBCs in the lumen were observed

**Fig. 2:**
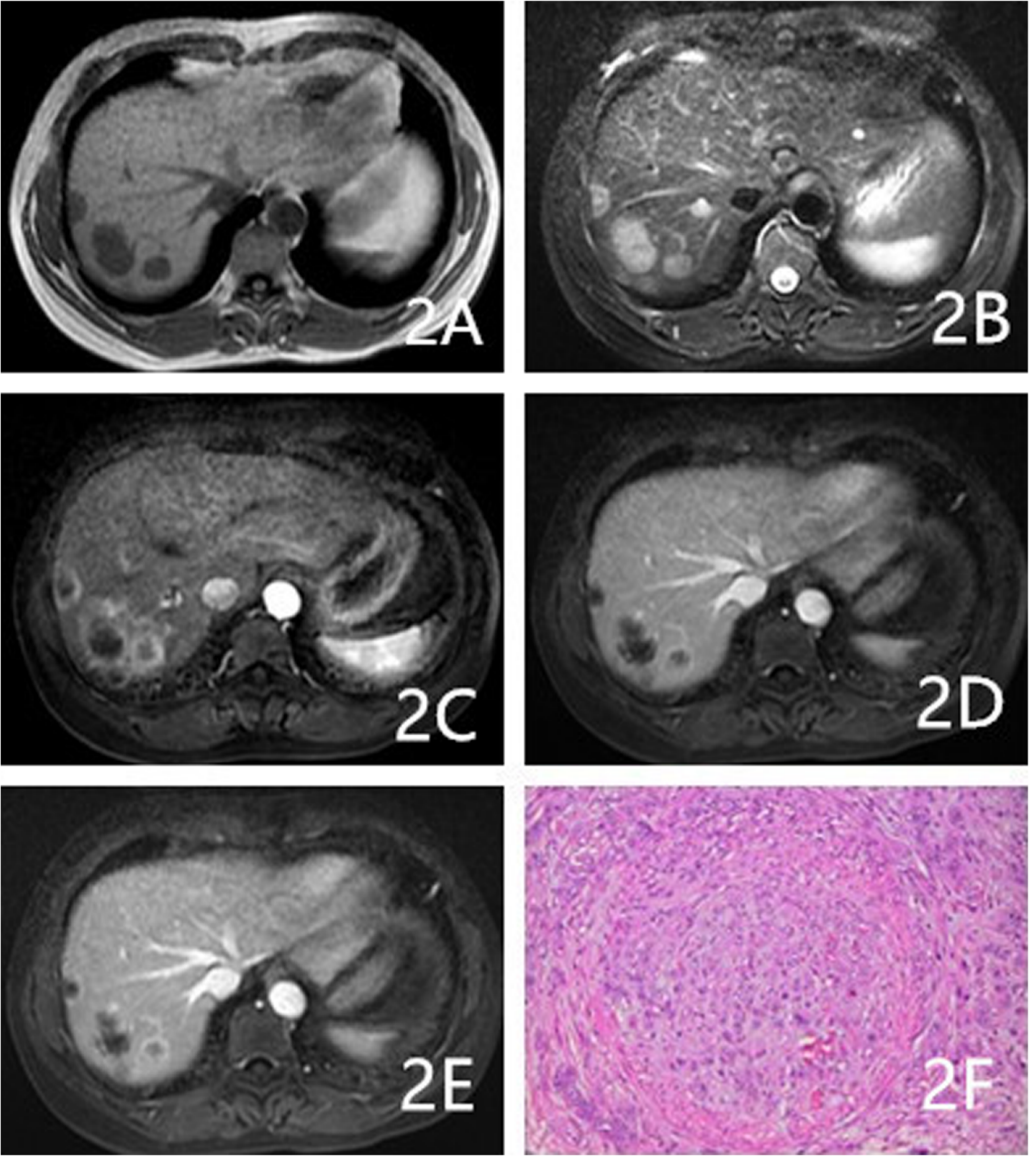
A 53-year-old female had multiple HEHE lesions in the right hepatic lobe. **a**-**b**: Multiple homogeneous signal masses were observed on T1WI and T2WI. **c**-**e**: The enhanced scan showed rim enhancement in the arterial phase and progressive centripetal enhancement in the portal and delayed phases. **f**: Magnification 200×. Epithelium-like tumor cells were closely arranged. A few lumens and RBCs in the lumen were observed

**Fig. 3:**
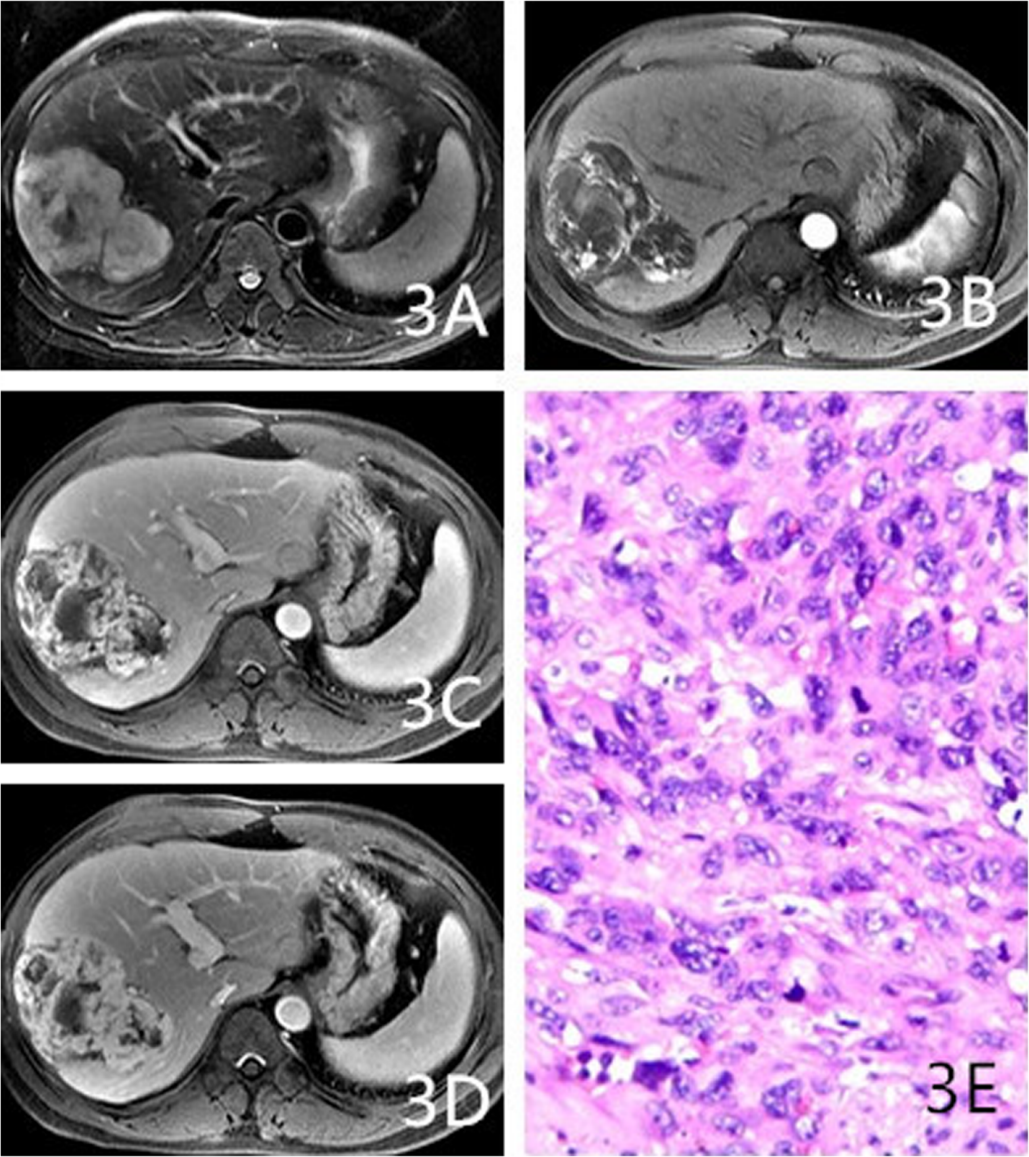
A 36-year-old male had right HAS. **a**: Heterogeneous high signal T2WI masses were observed. **b**-**d**: Enhanced scans of the masses showed centripetal enhancement with heterogeneous filling in each phase. **e**: Magnification 200×. The tumor cells were irregular in shape with dark-stained nuclei showing mitotic figures

**Fig. 4:**
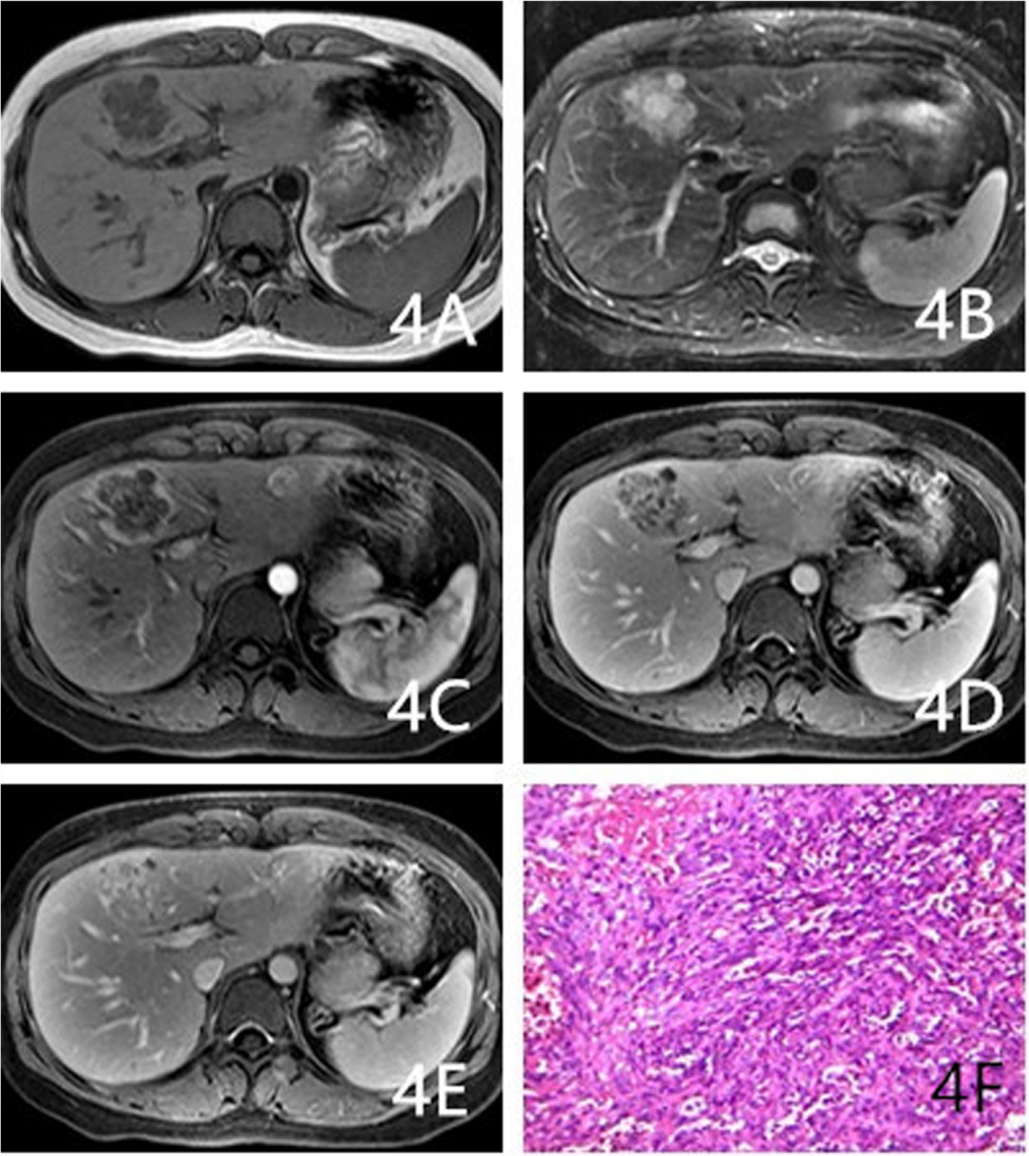
A 68-year-old female had left HAS. **a-b**: Multiple heterogeneous signal masses were observed on T1WI and T2WI. **c-e**: Enhanced scans of the mass showed centripetal enhancement with heterogeneous filling in each phase. **f**: Magnification 200×. Irregularly arranged lumens, large dark-stained nuclei, and flaky hemorrhages were observed

As Table 2 shows, the proportions of cases with circumscribed margins were 88% (22/25), 84.6% (11/13), and 31.6% (6/19) for hemangioma, HEHE, and HAS, respectively (*P* < 0.001). The differences among these groups were significant, and HAS lesions were more likely to not have circumscribed margins. The proportions of lesions with hemorrhaging were 4% (1/25), 30.8% (4/13), and 36.8% (7/19) for hemangioma, HEHE, and HAS, respectively, (*P* = 0.014). The proportions of cases with homogeneously low T1WI signals were 72% (18/25), 46.1% (6/13), and 21% (4/19) for hemangioma, HEHE, and HAS, respectively (*P* = 0.004). HEHE and HAS were more likely to show heterogeneous signals on T1WI (Figs. 1a, 3a, and 4a). The proportions of cases with homogeneously high signals on T2WI 64% (16/25), 23.1% (3/13; Fig. 2b), and 10.5% (2/19) for hemangioma, HEHE, and HAS, respectively (*P* = 0.001). HEHE and HAS were more likely to show heterogeneous signals on T2WI. Centripetal enhancement was the common pattern in vascular tumors, with proportions of 100, 46.2% (6/13), and 68.4% (13/19) for hemangioma, HEHE, and HAS, respectively (Figs. 3b-d and 4c-e). The difference in the enhancement pattern between the HEHE and HAS groups was significant(*P* < 0.001), and rim enhancement was common in cases of HEHE (46.2%, 6/13; Figs. 1c-e, 2c-e). In addition, three cases of HAS (15.8%, 3/19) showed no enhancement in any of the phases.

### Microscopic observations

Hemangiomas showed tumor tissues composed of many thin-walled and anastomosing blood vessel lumens. The lumens contained blood, and the vessel walls were vascular endothelial cell monolayers. Cells were typical, with local interstitial hyperplasia and blood vessel deformation due to compression.

In the HEHE group, the tumor tissue centers showed large collagen connective tissues and mucus matrices, with many epithelioid tumor cells (some of which were imprinted-like) and small vascular lumens rich in red blood cells (RBCs). In the tumor tissue, a few residual hepatocyte cords and small bile ducts were observed, with no capsule in the periphery area. The hepatic sinusoids in the neighboring liver tissues showed tumor cell growth, and the hepatic lobule structure was normal. Capsules were observed on the edges of a few HEHE cases (2/13, 15.4%). The immunohistochemistry results were as follows: HeP-1(−), CK18(+), CK19(−), CD24(−), β-Catenin(+), CA199(−), P53(+), CD31(+), and CD34(+).

In the HAS group, tumor cells were solid and arranged in a fissure-like structure, forming a local vascular cavity-like structure. The lumens were irregular and anastomosing. Some vascular lumens were broken and invaded the liver tissue with large hemorrhages. Tumor cells were obviously atypical, showing pathological nuclear mitotic figures. The immunohistochemistry results were as follows: CD31(+), CD34(+), Vimentin(+), CK(−), CK8(−), CK7(−), hepatocyte(−), AFP(−), Actin(−), Desmin(−), and S-100(−).

## Discussion

Hemangioma is common in vascular endothelial cell-derived benign and malignant tumors. Malignant tumors, including HEHE and HAS, are extremely rare. The incidence of HEHE is approximately 1 in 1 million [14], and 57% of patients are female [6]. In this study, the male:female ratio was 3:4, which approximates that reported in the literature. Few cases of HEHE present with jaundice or abnormal liver function, which might be caused when normal liver tissue is replaced by tumor tissue [15]. Carcinoembryonic antigen (CEA) can be elevated with normal alpha-fetoprotein (AFP) and cancer antigen (CA)-199 [16]. Liver function and CEA were normal in the HEHE group, although AFP was elevated in one case of HEHE with hepatocellular carcinoma. The metastatic rate was 27–45.1%, and the most common metastasis sites were the lungs and bone [6, 17, 18]. Because of its low malignancy, the prognosis of HEHE is better than that of other liver malignancies, with a 5-year survival rate of 55.5% [19]. HEHE treatments include liver resection and liver transplantation [4, 16]. The clinical manifestations of HEHE are inconsistent with its histological characteristics. Its biological behavior and clinical outcomes cannot be predicted by the infiltration of the adjacent liver tissue, nuclear pleomorphisms, or mitotic count.

HAS is the most common interstitial liver malignancy, accounting for < 1% of primary liver tumors [3, 5]. The incidence in males is usually higher than that in females. HAS is highly invasive and has a poor prognosis. Even after surgical resection, most patients died within a year [2, 20]. The clinical data from the cases in this study were comprehensively analyzed, and the results showed that the patients with hemangioma, HEHE, and HAS had no history of toxic exposure. Platelet (PLT) counts were elevated in two HEHE cases and decreased in five HAS cases. In addition, coagulatory function was decreased in six HAS cases, and the rate for cases with increased AFP in the HAS group was higher than that for HEHE, possibly because the hepatocytes in the HAS group were more severely invaded, and cell regeneration was more frequent. Local or systemic coagulopathy is related to excessive platelet retention in poorly growing and differentiated vascular cavity and sinusoids [21]. Frequent intratumoral hemorrhaging can also consume many coagulation factors. Because of the rapid progression in the HAS group, metastases in other organs (e.g., the spleen and lungs) often occurred at diagnosis, and the surgical resection rate was lower than those of the hemangioma and HEHE groups.

The proportion of cases without circumscribed lesion margins on MRI in the HAS group was significantly higher than those in the hemangioma and HEHE groups because of the aggressiveness and rapid peripheral invasion of HAS. A significant difference was also noted in the hemorrhage rate among the three groups. Higher malignancy degrees were associated with complicated intratumoral hemorrhaging, because the higher the degree of malignancy, the more discontinuous the blood vessels, the more prone to bleeding. The HEHE lesions contained numerous collagen connective and fibrous tissue, and hemorrhaging with necrosis can easily occur in patients with HAS, thereby resulting in heterogeneous signals on MRI plain scans. Hemangiomas exhibited typical centripetal enhancement, regardless of size or number of lesions. The contrast agent can fill the entire lesion, which corresponds to the rich and complete vascular network in the microscopic tumor tissue without significant necrosis or hemorrhaging. Because HEHE is associated with a large amount of collagen and fibrous connective tissue, patients’ epithelium-like tumor cells and tumor lumens were dense at the edges of some lesions, whereas others were associated with staggered connective tissues and tumor blood vessel lumens with hemorrhaging and necrosis in areas. Thus, the 13 HEHE cases in this study included six cases of edge enhancement and six cases of centripetal heterogeneity. HAS is not associated with connective tissue. Because of its high degree of tumor cell malignancy, poor differentiation, discontinuous vascular networks, and many hemorrhage sites, most cases (13/19) showed heterogeneous centripetal enhancement. Of the three HAS lesions with the worst tumor cell differentiation, only the scattered vascular endothelial cells were observed to float on the blood pool under a microscope, without MRI enhancement.

Differential diagnosis. HEHE and HAS with rim enhancement and heterogeneous centripetal enhancement should be differentiated from cholangiocarcinoma of the liver. Intrahepatic masses of cholangiocarcinoma are rich in fibrous tissue at the lesion center, with fewer peripheral fibers, and they often manifest with progressive and centripetal enhancement. Three typical enhancements have been reported [22]: 1) edge enhancement during the arterial phase with delayed filling; 2) heterogeneous enhancement during the early arterial phase with continuous enhancement during the delayed phase; and 3) slight peripheral enhancement during the early arterial phase with an unfilled center during the delayed phase. Indirect signs include liver capsule retraction and hepatic vein embedding. If a tumor embolus exists through the portal vein, then it can also be used to distinguish cholangiocellular carcinoma from HEHE and HAS. Sarcoma tumor cells adhere poorly and rarely to blood vessels to form nests.

This study has limitations. Because it is a retrospective and multicenter study, specific scanning parameter settings of the vascular tumors do not exist, and no DWI sequence data could be collected. In the future, we hope to cooperate with more hospitals to collect more vascular tumor clinical and imaging data for comparative analyses, especially for preliminary apparent diffusion coefficient (ADC) value comparisons of these tumors.

## Conclusions

In conclusion, we found many differences in the MRI signs of the three types of vascular tumors. Higher degrees of malignancy were associated with more blurred lesion boundaries, greater likelihoods of combined bleeding, and more uneven signals. However, little difference was found regarding the enhancement mode of the enhanced scan. However, HAS and HEHE did not significantly differ from benign hemangiomas in terms of age of onset, sex, lesion size, lesion number, PLT, coagulation function, AFP, or CEA. The 5-year survival rate for HAS was significantly lower than that of HEHE.

## Data Availability

The data needed to replicate the current findings are available in the figures and tables of the main article. Because of patient privacy protection, additional study materials are only available upon individual request directed to the corresponding author.

## Abbreviations

HEHE: Hepatic epithelioid hemangioendothelioma
HAS: Hepatic angiosarcoma
MRI: Magnetic resonance imaging
CT: Computed tomography
T1WI: T1-weighted imaging
T2WI: T2-weighted imaging
